# Production of Plasmatic Cell Juices—Experiments in Immunization and Clinical Treatment with the Plasmatic Cell Juices of Bacteria

**Published:** 1898-05

**Authors:** 


### Production of Plasmatic Cell Juices—Experiments in Immunization and Clinical Treatment with the Plasmatic Cell Juices of Bacteria.

Both of these valuable papers—the former by Prof. Hans Buchner; the latter by Dr. Martin Hahn—appear in the *Munchener Medicinische Wochenschrift*, 1897, No. 48. Prof. Buchner reports further on his method of obtaining the plasmatic cell contents, without recourse to chemical action by the mechanical trituration of the moist germ mass, followed by expression of the magma thus obtained in a hydraulic press at 400 to 1500 atmospheres. This method was first applied to yeast cells, and Buchner thus obtained a clear yellow, slightly opalescent liquid possessing a very considerable proportion of albumin. This liquid was shown by E. Buchner to be capable of producing genuine alcoholic fermentation in the absence and without the co-operation of any living organisms whatsoever. The real depository of the fermentative action is therefore a peculiar enzyme-like substance which is also capable of acting independently of the living cell and which received the name Zymase.

In moist condition this substance readily undergoes alteration; the fermentative properties also disappear spontaneously on somewhat prolonged storage; and this disappearance has probably some connection with the existence of powerful digestive enzymes observed by Dr. Hahn in the expressed juices, these enzymes giving rise to a species of auto-digestion. On the other hand, dry Zymase is permanent.

The next step was naturally the production by the same method of the expressed juices of pathogenic bacteria with a view to studying their specific properties. The manufacture of these bodies, to which Buchner gives the name Plasmins, presupposes the dispelling of technical and biological difficulties. This task was assumed by Dr. Hahn, and the second paper mentioned above contains his results.

He experimented with three types of pathogenic bacteria: 1, the cholera or typhus bacilli, which in guinea-pigs produce only acute and local infection; 2, anthrax bacilli or staphylococci, which give rise to acute, general infection; and, 3, tubercle-bacilli, which provoke chronic general infection.

The juice obtained by expressing cholera bacilli (cholera-plasmin) is strongly albuminous, the albumin behaving like a neuclo-albumin. To guinea-pigs it is toxic in a very limited degree, the pigs being killed only by larger doses; the local action consists in an inflammatory infiltration. It is easy to immunize guinea-pigs, with the aid of the choleraplasmin against peritoneal infection with living cholera bacteria, either by repeated small doses or by large doses given at one time. This immunization is strictly specific and persists for three or four months. The destruction of the cholera vibrionea in the organism of the animals immunized with the expressed juice, proceeds amid the symptoms observed by Pfeiffer; and yet not only the exudate, but also the blood serum of these animals, possessed specific agglutinating properties; very similar to the foregoing were the results with the typhoplasmin.

Hahn does not believe that there is any field for the therapeutic use of choleraplasmin with human beings. At the most it could be used only for prophylactic injections in the sense of Haffkine's experience.

The typhoplasmin, on the other hand, could not be used for therapeutic as well as immunizing purposes; nevertheless, it seems questionable whether immunity against peritoneal infection is identical with that against an intestinal disorder.

The experiments with the expressed juices of anthrax bacilli and staphylococci have shown that it will scarcely be possible to achieve, with their aid, a sure immunization against general infection. Though the animals treated succumbed somewhat later than the control animals, this fact could be explained by the elevation of bactericidal properties due to hyperleucocytosis.

With the expressed juices of tubercle-bacilli (which give promise of practical results) Dr. Hahn made his experiments six months before the appearance of Koch's publication. These experiments have not yet been concluded and require to be supplemented by clinical experiments on human beings. The tuberculoplasmin is a clear, amber-yellow liquid, containing much coagulable albumin; decomposes hydrogen peroxide (in contradistinction to Koch's new tuberculin); and may be stored for a considerable time in an ice-chest without the development of germs, by the addition of 30 per cent. glycerine and 5 per cent. common salt. With this preparation Hahn treated a number of guinea-pigs. Two weeks after innoculation he began injecting very small, gradually-augumented doses, which produced moderate but distinct symptoms of fever, the injections being prolonged for months. Of the seventeen guinea-pigs thus treated, three died before there was any possibility of a curative action; five others succumbed, in common with the control animals; but with four other guinea-pigs there was visible, despite the fact that death was not prevented, an anatomically lesser distribution or a reactive modification in the vicinity of the tubercle. The remaining five animals have thus far survived the control pigs one and a half to two months. Thus, almost one-third of the series were preserved, and in view of the inborn susceptibility of guinea-pigs to the tubercle-bacillus, this may be considered a not unfavorable result.

Investigations with the human subject would seem in order, especially as clinical tests thus far made have demonstrated the harmlessness of the remedy in human therapy, inasmuch as the patients are commonly presented for treatment in an advanced stage, and are complicated by secondary infections; and since it is not possible to inject into a human being a quantity proportionate to that given the test animal. But on the other hand, some benefit could be derived from the non-specific power of the tuberculoplasmin to produce hyperleucocytosis, where favorable influence on experimental infections has been repeatedly emphasized.—*Abstract in the Therapeutische Monatshefto*, January, 1898.

